# Overcoming the Intrinsic Difference between Hydrophilic CH_3_NH_3_PbI_3_ and Hydrophobic C_60_ Thin Films to Improve the Photovoltaic Performance

**DOI:** 10.3390/nano7070166

**Published:** 2017-07-03

**Authors:** Lung-Chien Chen, Yu-Shiang Lin, Zong-Liang Tseng, Chiale Wu, Feng-Sheng Kao, Sheng-Hui Chen

**Affiliations:** 1 Department of Electro-Optical Engineering, National Taipei University of Technology, Taipei 10608, Taiwan; t104658044@ntut.edu.tw (Y.-S.L.); tw78787788@yahoo.com.tw (Z.-L.T.); 2 Materials and Chemical Research Laboratories, Industrial Technology Research Institute, Hsinchu 31040, Taiwan; ChiaLeWu@itri.org.tw (C.W.); FSKao@itri.org.tw (F.-S.K.); 3 Department of Optics and Photonics, National Central University, Taoyuan 32001, Taiwan; ericchen@dop.ncu.edu.tw

**Keywords:** CH_3_NH_3_PbI_3_, wettability, C_60_, photovoltaics

## Abstract

Dimethylformamide/dimethyl sulfoxide solvent mixtures were used as the CH_3_NH_3_PbI_3_ (MAPbI_3_) precursor solvent in a one-step spin coating method to fabricate smooth and hydrophilic crystalline MAPbI_3_ thin films on top of hydrophobic carbon-60 (C_60_) thin film for highly efficient photovoltaics. The structural, optical, and excitonic characteristics of the resultant MAPbI_3_ thin films were analyzed using X-ray diffraction (XRD), atomic-force microscopy, absorbance spectroscopy, photoluminescence (PL) spectrometry, and nanosecond time-resolved PL. There was a trade-off between the crystallinity and surface roughness of the MAPbI_3_ thin films, which strongly influenced the device performance of MAPbI_3_-based photovoltaics. The high power conversion efficiency (*PCE*) of 17.55% was achieved by improving the wettability of MAPbI_3_ precursor solutions on top of the C_60_ thin films. In addition, it was predicted that the fill factor and *PCE* could be further improved by increasing the crystallinity of the MAPbI_3_ thin film while keeping it smooth.

## 1. Introduction

High-quality organic lead halide perovskites (CH_3_NH_3_PbI_3_, CH_3_NH_3_PbI_3−*x*_Cl*_x_*, and CH(NH_2_)_2_PbI_3_), which can be fabricated using a two-step or a one-step spin-coating method [1,2,3,4], have been widely used as the light harvesting material in photovoltaic cells due to their high power conversion efficiency (*PCE*) and low-cost of fabrication. It is well known that the low absorption bandgap (<1.6 eV) [5], small exciton binding energy (2–70 meV) [6,7,8,9], long exciton lifetime (>10 ns) [10,11,12], high carrier mobility (>5 cm^2^/Vs) [13,14], and long carrier diffusion length (>1 μm) [15,16] of perovskite thin films are the reasons why high-performance photovoltaic cells can be realized. The first use of CH_3_NH_3_PbI_3_ (MAPbI_3_) as a light harvesting material deposited on top of a hydrophilic mesoporous TiO_2_ film [17], resulted in a moderate *PCE* of 3.81% [18]. To date, perovskite-based photovoltaics with more than 15% *PCE* can be divided into two different types of structure: a regular-type photovoltaic structure [19], or an inverted-type photovoltaic structure [20], and the highest reported *PCE*s were 22.1% [21], and 19.0% [22], respectively. The device performance of perovskite-based photovoltaics is highly related to the crystallinity and thin-film continuity of the perovskite thin films. Smooth crystalline MAPbI_3_ thin films were first fabricated using a two-step solution process [1] with a porous PbI_2_ thin film used for the substrate, and a MAI thin film deposited on top of PbI_2_. After thermal annealing at ~100 °C for several hours in a nitrogen-filled glove box, a high-quality MAPbI_3_ thin film can be fabricated on top of a hydrophilic poly(3,4-ethylenedioxythiophene) polystyrene sulfonate (PEDOT:PSS) thin film through the inter-diffusion process [23]. Therefore, the thin-film quality of the resultant MAPbI_3_ perovskites fabricated by the two-step solution method is related to the fabrication environment [24]. High-quality MAPbI_3_ thin films can also be obtained using a one-step solution process with an in situ nonpolar antisolvent washing treatment [3,4,9]. Toluene, chlorobenzene, boromobenzene, and iodobenzene have been used as the anti-solvent in the washing treatment process while a mixture of gamma-butyrolactone (GBL) and dimethyl sulfoxide (DMSO), or a mixture of dimethylformamide (DMF) and DMSO was used as the MAPbI_3_ precursor solvent. The role of the antisolvent washing treatment was to increase the thin-film continuity of MAPbI_3_, with an increase in the number of nucleation sites and a reduction in crystallinity [25]. The selection of the MAPbI_3_ precursor solvent does not only determine the solubility, but also influences the wettability of the precursor solution on a substrate. For hydrophobic substrates (dense TiO_2_ and thermally evaporated C_60_ thin films), the use of DMF as the precursor solvent was due to good wettability. However, the crystallinity of the resultant thin films was not high when DMF was used as the precursor solvent as it has a low boiling point (BP) of 153 °C. It was predicted that the addition of DMSO (BP = 189 °C) in the MAPbI_3_ precursor solvent helped the crystal growth of MAPbI_3_ thin films during the thermal annealing process. However, the roles of DMF and DMSO in the MAPbI_3_ precursor for regular-type perovskite photovoltaics were not completely understood in previous reports [26,27,28,29]. The aim of this study was to explore the interplay between nucleation and crystal growth during the formation of MAPbI_3_ thin films, and to investigate the influence of the properties (wettability and boiling point) of the precursor solvent on high-performance MAPbI_3_-based photovoltaics. In this study, various volume ratios of DMF to DMSO were used as the MAPbI_3_ precursor solvent.

## 2. Experimental Section

C_60_ thin film was deopsited on top of an ITO/glass substrate with a sheet resistance of 7 Ω/sq using thermal evaporation at a rate of 0.01–0.02 nm/s under a high vaccum environment (1.5 × 10^−6^ torr). Next, 289 mg PbI_2_ (Sigma-Aldrich, Saint Louis, MO, USA, 99.9995%) and 98 mg MAI powders were dissoved in 500 μL DMF/DMSO solvent mixtures as the MAPbI_3_ precursor solution. MAI (Lumtec, Hsinchu, Taiwan, ROC, 98.5%) was purified by a solvent (diethyl ether and ethanol) to obtain pure MAI as white crystals [30]. The toluene-assisted one-step solution process was used to fabricate the MAPbI_3_ thin film on top of the C_60_/ITO/glass, and a detailed description of the fabrication process of MAPbI_3_ thin films has been presented in previous reports [3,4]. A Spiro-OMeTAD/chlorobenzens (40 mg/0.5 mL) solution with additives containing 8 μL Li-TFSI (Ruilong, Miaoli, Taiwan, ROC)/acetonitrile and 14.25 μL TBP (Ruilong, Miaoli, Taiwan, ROC) was spin coated on top of the MAPbI_3_/C_60_/FTO/glass as the hole transporting layer (HTL). Then, an Ag film was thermally evaporated onto the sample to act as the anode. The active area (0.1 cm^2^) of the photovoltaic cell was defined using a shadow mask during Ag evaporation, where the resultant photovoltaic structure was comprised of Ag/Sprio-OMeTAD/MAPbI_3_/C_60_/ITO/glass. The thicknesses of ITO (Ruilong, Miaoli, Taiwan, ROC), C_60_, MAPbI_3_, Spiro-OMeTAD (Ruilong, Miaoli, Taiwan, ROC), and Ag were controlled at ca. 170 nm, 15 nm, 480 nm, 125 nm, and 100 nm, respectively. The current density-voltage (*J*-*V*) curves of the photovoltaic cells were obtained using a source-measurement unit (Keithly, Cleveland, HO, USA, 2400). The optical intensity of the simulated sunlight was calibrated using a reference cell (Oriel, Strarford, CT, USA, 91150V) with an optical filter (KG-5) to have an intensity of 100 mW/cm^2^. The contact angles of the C_60_ thin film were measured using a home-made image measurment system. The surface morphologies of MAPbI_3_ thin films were measured using a contact-mode atomic-force microscope. The absorbance spectra were determined by a visible spectrometer. The crystallinities of MAPbI_3_ thin films were determined by a commercial X-ray diffractormeter (PANalytical, Almelo, Netherlands, PW-1830). The photoluminescence (PL) spectrometer (Protrustech, New Taipei City, Taiwan, ROC) and nanosecond time-resolved PL were measured by a commerical optical microscope-based detection system.

## 3. Results and Discussion

**Figure 1:**
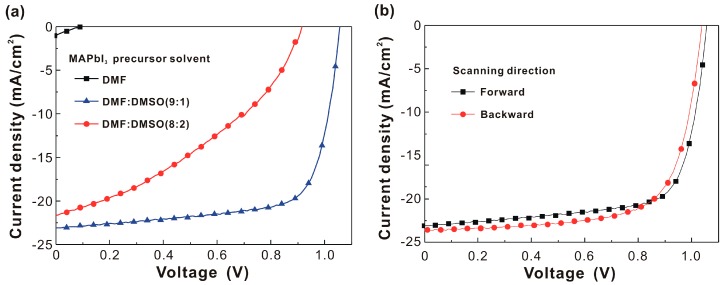
(**a**) *J*-*V* curves of MAPbI_3_-based photovoltaics under one sun illumination under forward scanning direction; (**b**) *J*-*V* curves of the best MAPbI_3_-based photovoltaics under one sun illumination under forward and backward scanning directions.

The *J*-*V* curves under a forward scanning direction for the MAPbI_3_-based solar cells are presented in Figure 1a. The average photovoltaic performance of 16 devices for each fabrication condition is summarized in Table 1. With an increase in the volume ratio of DMSO to DMF from 0/10 to 1/9, the averaged open-circuit voltage (*V*_OC_), short-circuit current density (*J*_SC_), and fill factor (*FF*) of the MAPbI_3_-based photovoltaics dramatically increased from 0.108 V, 0.84 mA/cm^2^, and 22.9% to 1.045 V, 22.10 mA/cm^2^, and 70.1%, respectively. This implied that the addition of DMSO in the MAPbI_3_ precursor changed the properties of the resultant MAPbI_3_ thin films. The averaged *FF* (*PCE*) of the MAPbI_3_-based photovoltaics significantly decreased from 70.1% (16.21%) to 39.2% (7.80%) with an increase in the volume ratio of DMSO to DMF from 1/9 to 2/8. The reduction in the *FF* from 70.1% to 39.2% was due to the reduced shunt resistance and the increased series resistance, which suggested poor interfacial contacts [31]. To assess the hysteresis effect of MAPbI_3_-based photovoltaics, the *J*-*V* curves were measured under different scanning directions as shown in Figure 1b. The hysteresis in the *J*-*V* curves indicated that the *PCE* could be further increased when the origins of the hysteresis were diminished.

**Figure 2:**
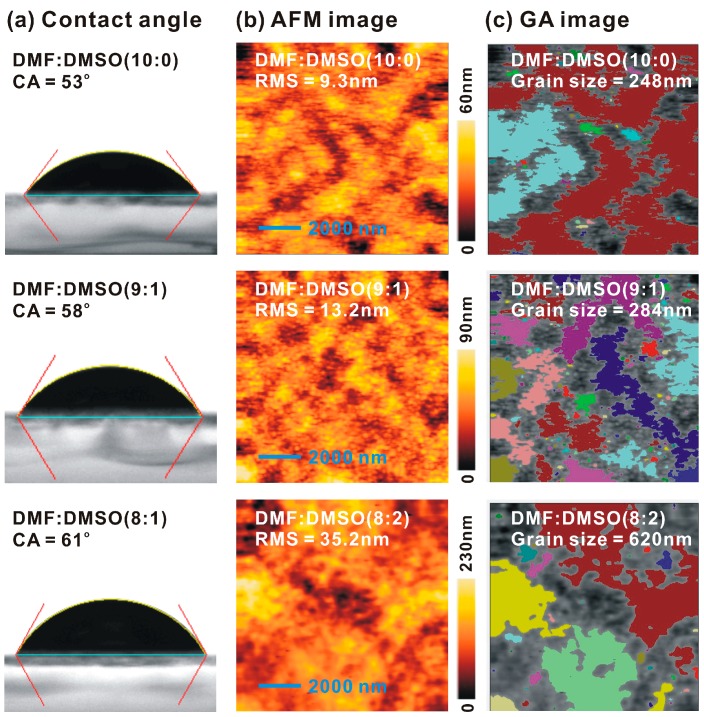
(**a**) Contact angles (CAs) of MAPbI_3_ precursor solutions on C_60_/ITO/glass samples; (**b**) Atomic force microscope (AFM) images of the MAPbI_3_/C_60_/ITO/glass samples; (**c**) Graphical analysis (GA) images of the corresponding AMF images. The standard deviations of the contact angles were smaller than 1°.

The droplet contact angles on the C_60_/ITO/glass sample, as presented in Figure 2a, indicate that the addition of DMSO decreased the wettability of the MAPbI_3_ precursor solutions on the C_60_ thin film. It has previously been demonstrated that larger contact angles on the substrate resulted in a crystalline perovskite thin film on the substrate due to the non-wetting surface-driven high-aspect-ratio crystalline grain growth [32]. Figure 2b presents the surface morphologies of the MAPbI_3_/C_60_/ITO/glass samples. The average grain sizes (see Figure 2c) and peak-to-valley heights of the MAPbI_3_ thin films were determined by the built-in graphical analysis tools in the AFM measurement system, where the trend of the grain sizes was proportional to the trend of the contact angles. Conceptually, the roughened MAPbI_3_ thin films with a large peak-to-valley height of 268 nm could not be completely covered with a 125 nm-thick Spiro-OMeTAD thin film. Therefore, the reduction in the *FF* from 70.1% to 39.2% was due to poor contact at the interface between the MAPbI_3_ and the Spiro-OMeTAD when the volume ratio of DMSO to DMF increased from 1/9 to 2/8.

**Figure 3:**
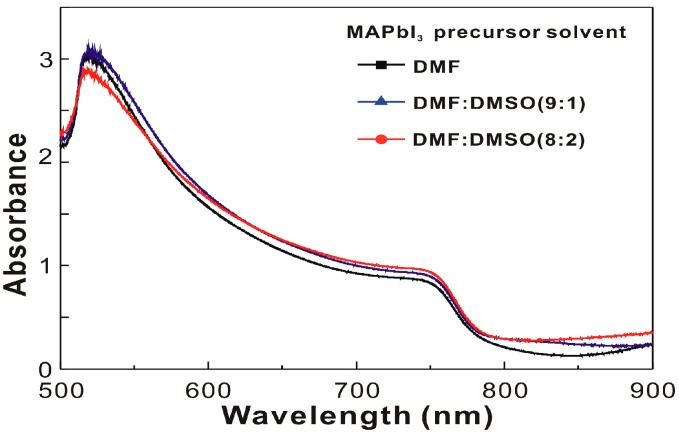
Absorbance spectra of MAPbI_3_/C_60_/ITO/glass samples.

Figure 3 presents the absorbance spectra of the MAPbI_3_/C_60_/ITO/glass samples. For 250 nm-thick MAPbI_3_ thin films, the amplitude of the ripple in the wavelength region from 800–900 nm can be used to assess the surface roughness of the MAPbI_3_ thin film as the amplitude trend of the ripple is inversely proportional to the surface roughness of the MAPbI_3_ thin film [33]. Furthermore, the addition of DMSO to the MAPbI_3_ precursor solution did not significantly influence the light harvesting ability in the resultant MAPbI_3_ thin films.

**Figure 4:**
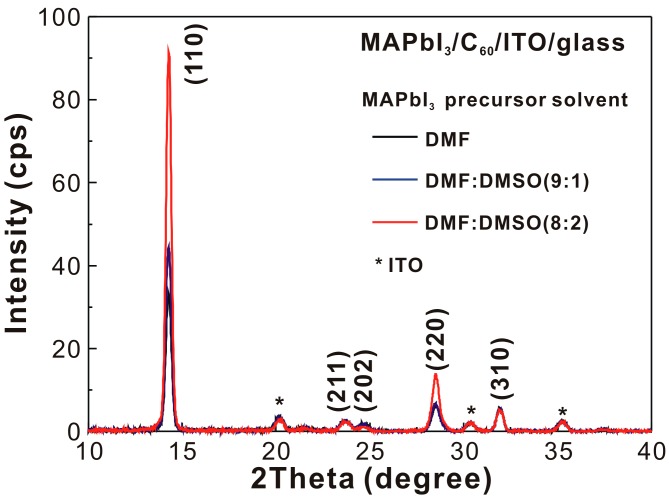
X-ray diffraction patterns of MAPbI_3_/C_60_/ITO/glass samples.

Figure 4 presents the X-ray diffraction patterns of the MAPbI_3_/C_60_/ITO/glass samples. The main diffraction peak (110) was used to calculate the crystal domain sizes of the MAPbI_3_ thin films with the Scherrer equation, which are listed in Table 2. Results indicated that the addition of DMSO in the MAPbI_3_ precursor solution did increase the crystallinity of the resultant MAPbI_3_ thin films.

**Figure 5:**
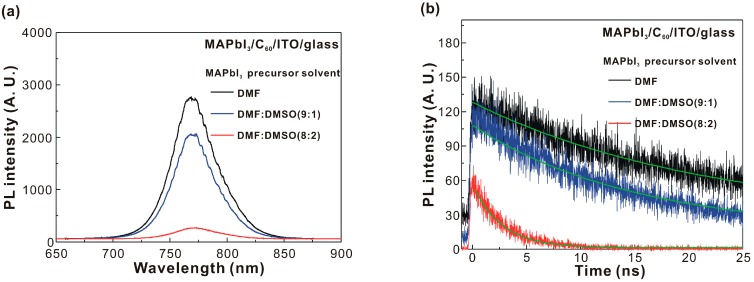
(**a**) Static photoluminescence spectra of MAPbI_3_ thin films on C_60_/ITO/glass; and (**b**) Nanosecond time-resolved photoluminescence of MAPbI_3_ thin films on C_60_/ITO/glass.

**Figure 6:**
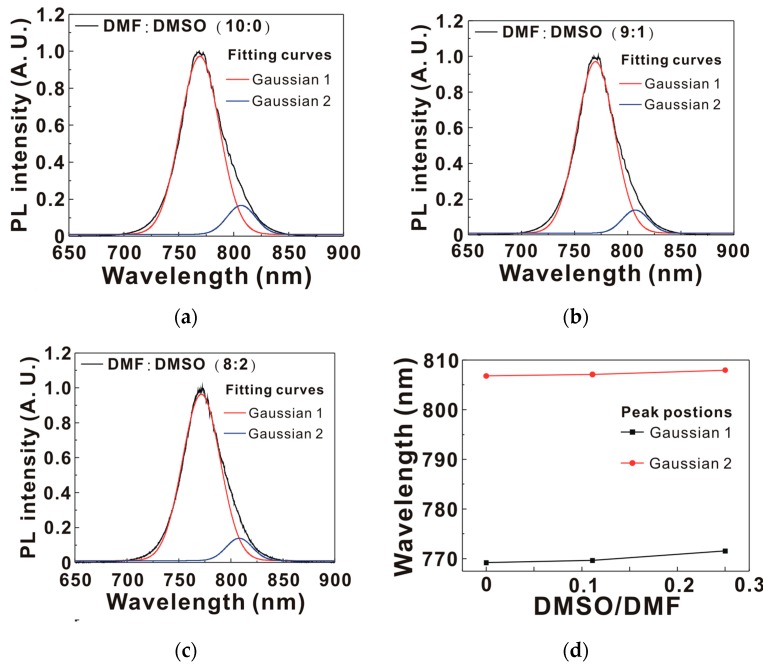
(**a**–**c**) The normalized PL spectra of the MAPbI_3_/C_60_/ITO/glass samples; (**d**) the peak emission wavelengths of the MAPbI_3_/C_60_/ITO/glass samples.

Figure 5a presents the PL spectra of MAPbI_3_/C_60_/ITO/glass samples. When the hot excitons relax to the conduction band from the excited state through rapid thermalization (downhill relaxation), about half of the excitons in the MAPbI_3_ thin film can self-dissociate at room temperature and form free carriers, due to the low exciton binding energies [6,7,8,9]. As the other half of the excitons have to diffuse to the p-n junction interface to generate free carriers, the PL intensity from the residual excitons in the MAPbI_3_ thin films can be used to evaluate the exciton dissociation at the interface. The addition of DMSO in the MAPbI_3_ precursor solution decreased the PL intensity of the MAPbI_3_ thin film, indicating that the PL quenching ability was proportional to the crystallinity of the MAPbI_3_ thin film. Figure 5b presents the exciton dynamics in the MAPbI_3_/C_60_/ITO/glass samples. The time-dependent PL intensity was fitted with an exponential decaying function to obtain the exciton lifetime (see Table 2) in the MAPbI_3_ thin film. The trend of the exciton lifetimes was consistent with the trend of the PL intensities, which indicated that PL quenching was due to exciton dissociation at the interface between the MAPbI_3_ thin film and C_60_ thin film. The decreased PL intensity and PL lifetime were not caused by the trap-assisted nonradiative recombination as the exciton lifetimes (or PL lifetimes) of the MAPbI_3_ thin films on C_60_/ITO/glass were shorter than the time (~100 ns) for the electrons to transfer from the conduction band to the trap level in the MAPbI_3_ thin films [34]. The addition of DMSO increased the exciton dissociation rate at the interface between the MAPbI_3_ and C_60_ thin films. To analyze the asymmetric characteristics in the PL spectrum, the normalized PL spectrum was fitted with the two Gaussian curves shown in Figure 6. There were two Gaussian peaks at the wavelengths of ~770 nm and ~807 nm, which were assigned to the radiative decays from the band-to-band transition and the trap-assisted transition of the MAPbI_3_ thin film [35], respectively. The fitting results indicated that the emission wavelengths of the two Gaussian peaks were both red-shifted when the addition of DMSO in the MAPbI_3_ precursor solution was increased. The peak emission wavelengths of the MAPbI_3_/C_60_/ITO/glass samples are listed in Table 2. The trend of the peak emission wavelengths was proportional to the trend of the crystal domain sizes of the MAPbI_3_ thin films. The intensity ratio of Gaussian 1 to Gaussian 2 (see Table 2) was used to assess the crystallinity of the corresponding MAPbI_3_ thin film. In addition, the trend of the peak positions of Gaussian 1 was proportional to the crystal domain sizes of the MAPbI_3_ thin films.

To deposit a hydrophilic MAPbI_3_ thin film on top of a hydrophobic C_60_ thin film, DMF was used as the solvent to increase the wetting force of the MAPbI_3_ precursor solution on C_60_ thin film, as shown in Figure 1a. The addition of DMSO to the MAPbI_3_ precursor solution increased the crystallinity of the MAPbI_3_ thin film, or in other words, the wettability of the DMF on the C_60_ thin film and the high boiling point of the DMSO (189 °C) increased the nucleation and crystal growth of the MAPbI_3_ thin film, respectively. Therefore, there was a trade-off between the thin-film continuity and crystallinity for the formation of a MAPbI_3_ thin film. The high *V*_OC_ (1.045 V) was the result of the difference between the Fermi level of the Spiro-OMeTAD thin film, and the Fermi level of the C_60_ thin film. The high *J*_SC_ (22.1 mA/cm^2^) was due to the large light-harvesting ability of the multi-crystalline MAPbI_3_ thin film. The high *FF* (70.1%) was explained as being due to the formation of a smooth crystalline MAPbI_3_ thin film, which was advantageous to the smooth contact between the MAPbI_3_ thin film and the Spiro-OMeTAD capping layer.

## 4. Conclusions

In summary, we fabricated a high performance C_60_-based CH_3_NH_3_PbI_3_ (MAPbI_3_) photovoltaic cell. The highest power conversion efficiency (*PCE*) was 17.55% (17.20%) under the forward (backward) scanning direction under one sun illumination. The high *PCE* relied on the formation of a smooth crystalline hydrophilic MAPbI_3_ thin film on a hydrophobic C_60_ thin film. By increasing the amount of DMSO added to the MAPbI_3_ precursor solution, the crystallinity and peak-to-valley height of the MAPbI_3_ thin film was increased. This occurred as a result of the increase in crystal growth time and a reduction in the number of nucleation sites, which resulted in an optimized photovoltaic performance when the volume ratio of DMSO to DMF was 1/9. The experimental results (atomic-force microscopic images and x-ray diffraction patterns) indicated that the 305-nm MAPbI_3_ grain was a multi-crystalline MAPbI_3_ particle with a crystal domain size of ~23 nm. Thus, it was possible to increase the *PCE* by increasing the crystallinity of the MAPbI_3_ thin film while keeping a low surface roughness. 

## Figures and Tables

**Table 1:** Photovoltaic performance under one sun illumination (AM 1.5G, 100 mW/cm^2^).

MAPbI_3_ Precursor Solvent	*V*_OC_ (V)	*J*_SC_ (mA/cm^2^)	*FF* (%)	*PCE* (%)
DMF	0.106 ± 0.028	0.84 ± 0.64	22.9 ± 0.5	0.02 ± 0.01
DMF:DMSO (9:1)	1.045 ± 0.011	22.10 ± 1.17	70.1 ± 3.2	16.21 ± 1.29
DMF:DMSO (9:1) ^a^	1.056	23.11	71.9	17.55
DMF:DMSO (9:1) ^b^	1.040	23.69	69.6	17.20
DMF:DMSO (8:2)	0.929 ± 0.032	21.58 ± 0.88	39.2 ± 5.2	7.80 ± 1.40

^a^ Photovoltaic performance of the best cell under the forward scanning direction; ^b^ Photovoltaic performance of the best cell under the backward scanning direction.

**Table 2:** Structural, optical, and excitonic properties of MAPbI_3_ thin films. PVH: peak-to-valley height; *D*: crystal domain size; *I*_XRD_: peak intensity of XRD at (110); PG1: peak position of Gaussian 1; PG2: peak position of Gaussian 2; Ratio: intensity ratio of Gaussian 1 to Gaussian 2; τ: exciton lifetime of MAPbI_3_ thin film on C_60_/ITO/glass sample.

MAPbI_3_ Precursor Solvent	PVH (nm)	*D* (nm)	*I*_XRD_	PG1 (nm)	PG2 (nm)	Ratio	τ (ns)
DMF	68	22.3	33	769.2	806.8	8.47	21.55
DMF:DMSO (9:1)	144	22.7	44	769.6	807.1	10.71	15.13
DMF:DMSO (8:2)	268	24.0	91	771.6	808.0	10.76	2.71
